# Isolation the bacteriophages against carbapenem- resistant *K. pneumoniae* (CRKP) from hospital wastewater effluent treatment in east-cost Malaysia

**DOI:** 10.1017/ash.2025.152

**Published:** 2025-09-03

**Authors:** Manal Abdel Haleem A. Abusalah, Chan Yean Yean, Aziah Ismail, Abdul Rahman Zaidah

**Affiliations:** 1 Department of Medical Microbiology & Parasitology, School of Medical SciencesUniversiti Sains Malaysia, 16150 Kubang Kerian, Kelantan, MALAYSIA; 2 Hospital Universiti Sains MalaysiaUniversiti Sains Malaysia, Kubang Kerian 16150, Kelantan, Malaysia; 3 Institute for Research in Molecular Medicine (INFORMM)Universiti Sains Malaysia, 16150 Kubang Kerian, Kelantan, MALAYSIA

**Keywords:** CRKP bacteriophage, MDR *K. pneumoniae*, phage isolation, sewage water

## Abstract

**Objectives:** Antibiotic resistance is a global issue that has significant negative effects on both health and the economy. Klebsiella pneumoniae is grouped with Enterococcus faecium, Staphylococcus aureus, Acinetobacter baumannii, Pseudomonas aeruginosa and Enterobacter spp. (ESKAPE) as multidrug-resistant (MDR) bacteria worldwide. The challenges associated with controlling life-threatening infections caused by MDR organisms have pushed research focus toward alternative treatments, which include bacteriophage therapy. Therefore, this study aimed to isolate the carbapenem-resistant K. pneumoniae (CRKP) specific phages from the hospital sewage water effluent for future application in a clinical setting. **Methods:** Sewage samples were obtained from different points of hospital effluent. The collected samples were primarily filtrated and centrifuged to recover, purify, and concentrate the bacteriophage. The lytic phages were detected using a spot assay. Subsequently, the specific CRKP phages were isolated using the double agar layer method, where the four CRKP clinical isolates were used as the host system. **Results:** Altogether, 30 sewage samples were collected from different points of hospital treatment plant at the Hospital Universiti Sains Malaysia (Hospital USM). Each samples were screened with four different clinical CRKP strains, giving rise to a total of 120 screened plates. Lytic phages were isolated in 50 /120 (41.70%) of the screened plates. The diameter of isolated CRKP lytic phages ranged between 0.01-0.7 cm. The phage titer ranged between 6×10^3^−1.6×10^9^ plaque- forming units per milliliter (PFU/ml). **Conclusion:** The lytic phages were isolated in abundance from the hospital treatment plant and exhibited a wide range of inhibitions against the CRKP, indicating its therapeutic potential in the future. However, further studies are required to comprehend the process of in vivo phage-mediated selection.

